# Structure and functions of the ventral tube of the clover springtail *Sminthurus viridis* (Collembola: Sminthuridae)

**DOI:** 10.1038/s41598-018-37354-4

**Published:** 2019-01-29

**Authors:** Chong-Guang Chen, Tong Chen, Bao-Zhen Hua, Tao-Ruan Wan

**Affiliations:** 1 Insect Laboratory, Gaoxin Campus, Xi’an Medical University, 168# West Part of South 2nd Ring Road, Xi’an, Shaanxi 710075 China; 2 College of Plant Protection, Northwest A&F University, Yangling, Shaanxi 712100 China; 3 Faculty of Engineering & Informatics, University of Bradford, West Yorkshire, BD7 1DP UK; 4 School of Life Sciences, Xinjiang Normal University, Urumqi, Xinjiang 830054 China

## Abstract

Springtails (Collembola) are unique in Hexapoda for bearing a ventral tube (collophore) on the first abdominal segment. Although numerous studies have been conducted on the functions of the ventral tube, its fine structure has not been thoroughly elucidated to date. In this paper, we observed the jumping behavior of the clover springtail *Sminthurus viridis* (Linnaeus, 1758) and dissected the ventral tube using light microscopy to elucidate the fine structure and the possible function of the ventral tube. The results show that a pair of eversible vesicles can be extended from the apical opening of the ventral tube. The eversible vesicles are furnished with numerous small papillae, and can be divided into a basal part and a distal part. The eversible vesicles have a central lumen connected to the tiny papillae and leading to the body cavity. The eversible vesicles can reach any part of the body, and may serve as following functions: (a) absorbing moisture; (b) uptaking water; (c) cleaning the body surface; and (d) fastening the body on a smooth surface.

## Introduction

Collembola are a primitive class in Hexapoda, and are among the most widespread and abundant terrestrial arthropods in the world^1^. Apart from the most characteristic feature of the jumping organ or furca, which evolved through the basal fusion of a pair of appendages on the fourth abdominal segment, all Collembola are characterized by the presence of a ventral tube, which is also called the collophore, on the ventral side of the first abdominal segment^1^. The ventral tube is presumed to represent the fused appendages of the first abdominal segment^2,3^. A pair of eversible vesicles is present on the distal end of the ventral tube^3^. Some scholars believe that the paired vesicles can be everted under hydraulic pressure from within the haemocoel^4–8^. The tips of vesicles carry suctorial cups enabling the extended tube to be used as a climbing aid when they crawl over smooth or steep surfaces^9^.

The functions of the ventral tube have been extensively studied^10–20^. This organ is extremely important in fluid and electrolyte balance, but can also function as a sticky appendage to enable springtails to adhere to slippery surfaces^1^. In some species, the vesicles of the ventral tube may extend more than twice the length of the body and be used for self-righting after a jump^21^. The eversible vesicles were regarded as a “cleaning” organ to clean up the body, or as a sticky appendage to enable springtails to adhere to slippery surfaces^1,2^. The adhesive ventral tube may even help direct the springtail jump^22,23^. An experiment carried out with *Sminthurus viridis* suggests that the ventral tube is used for conveying droplets of water from the hairs of the body to the mouth^24^.

The structure of the ventral tube differs among different orders^25^. The ventral tube is normally short in the order Poduromorpha, but is considerably long in the order Symphypleona, which is represented by the widespread family Sminthuridae^1^.

Representatives of Sminthuridae are among the most frequently encountered and brightly coloured springtails^26^. They live predominantly in superficial leaf litter, on low vegetation, or on the surface of still fresh water, and are abundant on trees, particularly in the canopies of tropical humid forests^23^. Sminthuridae have a characteristic globular shape formed by the enlargement and fusion of posterior thoracic and anterior abdominal segments^1^. Most species are exceptional jumpers and some have a conspicuous ventral tube, the vesicles of which when extruded extend for more than twice the length of the body^1,6^. Because the spherical abdominal cavity has a large volume, Sminthuridae can contain longer eversible vesicles in their globular abdominal cavity.

In this paper, we observed the clover springtail *Sminthurus viridis* (Linnaeus, 1758) and dissected the ventral tube using light microscopy to elucidate the fine structure and to explore the possible function of the ventral tube.

## Results

### Gross morphology of the eversible vesicles

The springtails are roughly spherical, around 1.00 mm in length. A short ventral tube is present on the ventral side of the first abdominal segment. We chose the living samples anesthetized with diethyl ether and soaked them in normal saline to dissect the worms under stereomicroscope.

**Figure 1:**
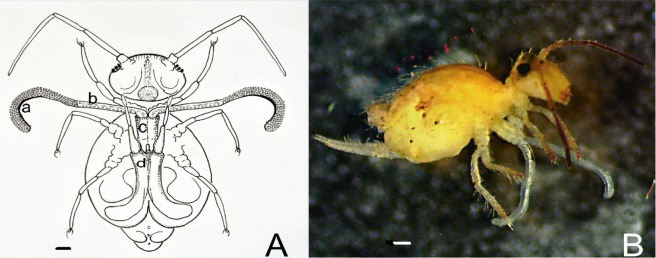
The eversible vesicles of *S. viridis*. (**A**) Schematic drawing of the adult in ventral view, showing the eversible vesicles out of the ventral tube. (a) Distal part of the eversible vesicle; (b) basal part of the eversible vesicle; (c) ventral tube; (d) furca; (**B**) lateral view of the adult, with the eversible vesicles extracted from the ventral tube. Scale bars = 0.05 mm.

**Figure 2:**
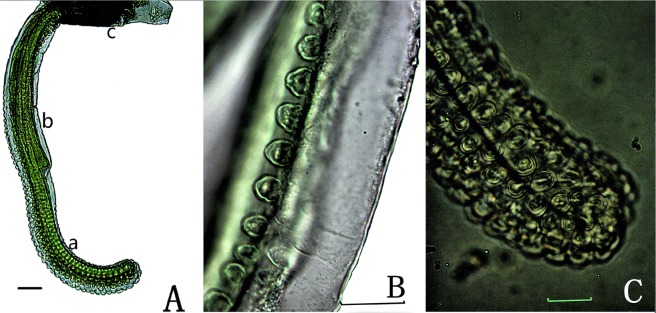
The eversible vesicles and small tiny papillae. (**A**) An eversible vesicle. (a) Distal part of the eversible vesicle; (b) basal part of the eversible vesicle; (c) the ventral tube; (**B**) small papillae on the basal part of the eversible vesicle; (**C**) small papillae on the distal part of the eversible vesicle. Scale bars = 0.02 mm.

**Figure 3:**
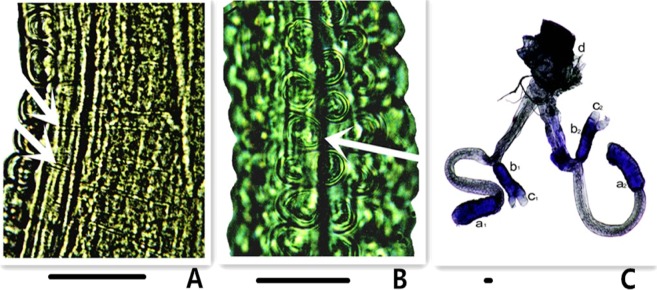
Liquid transmission duct of small papillae and the eversible vesicles. (**A**) The transverse duct (arrows) for connecting small papillae; (**B**) the distal part of the eversible vesicle, arrow pointing to the central lumen; (**C**) the eversible vesicles dissected out of the body, a1, a2, b1 and b2, muscle rods of the eversible vesicles; c1 and c2, interface to the digestive system; d, the ventral tube on the body surface. Scale bars = 0.02 mm.

A pair of eversible vesicles can be extended from the distal opening of the ventral tube (Fig. 1). The eversible vesicles are considerably long, and can extend out of the ventral tube to reach any part of the body. The eversible vesicles can be divided into a basal part and a distal part. The distal part is furnished with over 200 tiny papillae. A few tiny papillae are inserted in the basal part (Fig. 2). Each papilla has a transverse duct leading to the central lumen of the eversible vesicle (Fig. 3). The central lumen is connected to the body cavity, and is wrapped in the thick powerful muscular tissue. The eversible vesicles connect four muscle rods in the abdominal cavity (Fig. 3C). These muscle rods can stretch more than 10 times the length, and provide control to the activity of the eversible vesicles. The existence of papillae and their conveying ducts may help understand the functions of the eversible vesicles.

### The role and movement of the eversible vesicles

**Figure 4:**
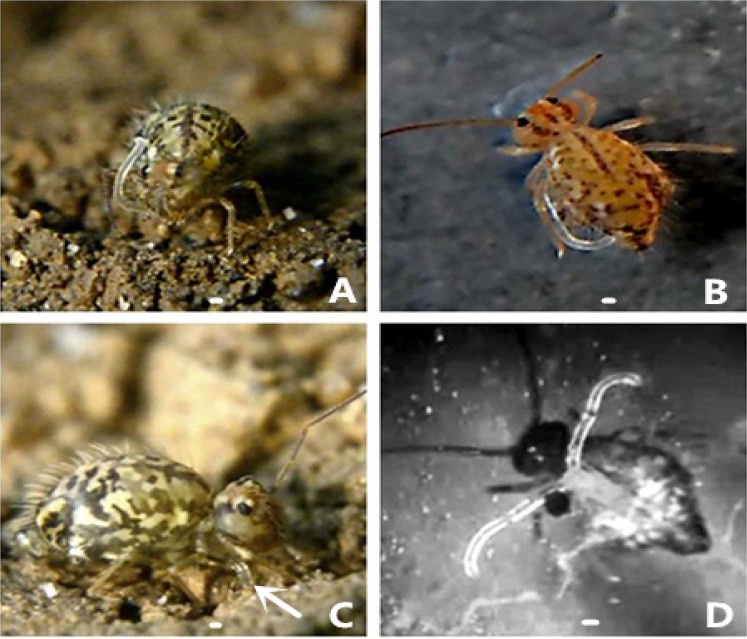
The springtail cleans the body with the eversible vesicles. (**A**) The eversible vesicle is cleaning the antennae; (**B**) the eversible vesicle is cleaning the tergum; (**C**) the springtail insert its eversible vesicles into the soil to absorb moisture; (**D**) in a test tube, the eversible vesicles help the body stick to the glass. Scale bars = 0.05 mm.

Anatomical experiments show that four retractable muscle rods are in the body (Fig. 3C). These muscle rods control the eversible vesicles, and help them to extend and retract rapidly. The eversible vesicles move very rapidly. For example, they usually spend less than three seconds to extend and retract the eversible vesicles when they clean the body (Fig. 4A,B). Sometimes the eversible vesicles took longer time to drink water. Due to the existence of the transverse duct and the central lumen, the small papillae can directly absorb liquid or adhere to the surface of a smooth substratum (Fig. 4C,D).

**Figure 5:**
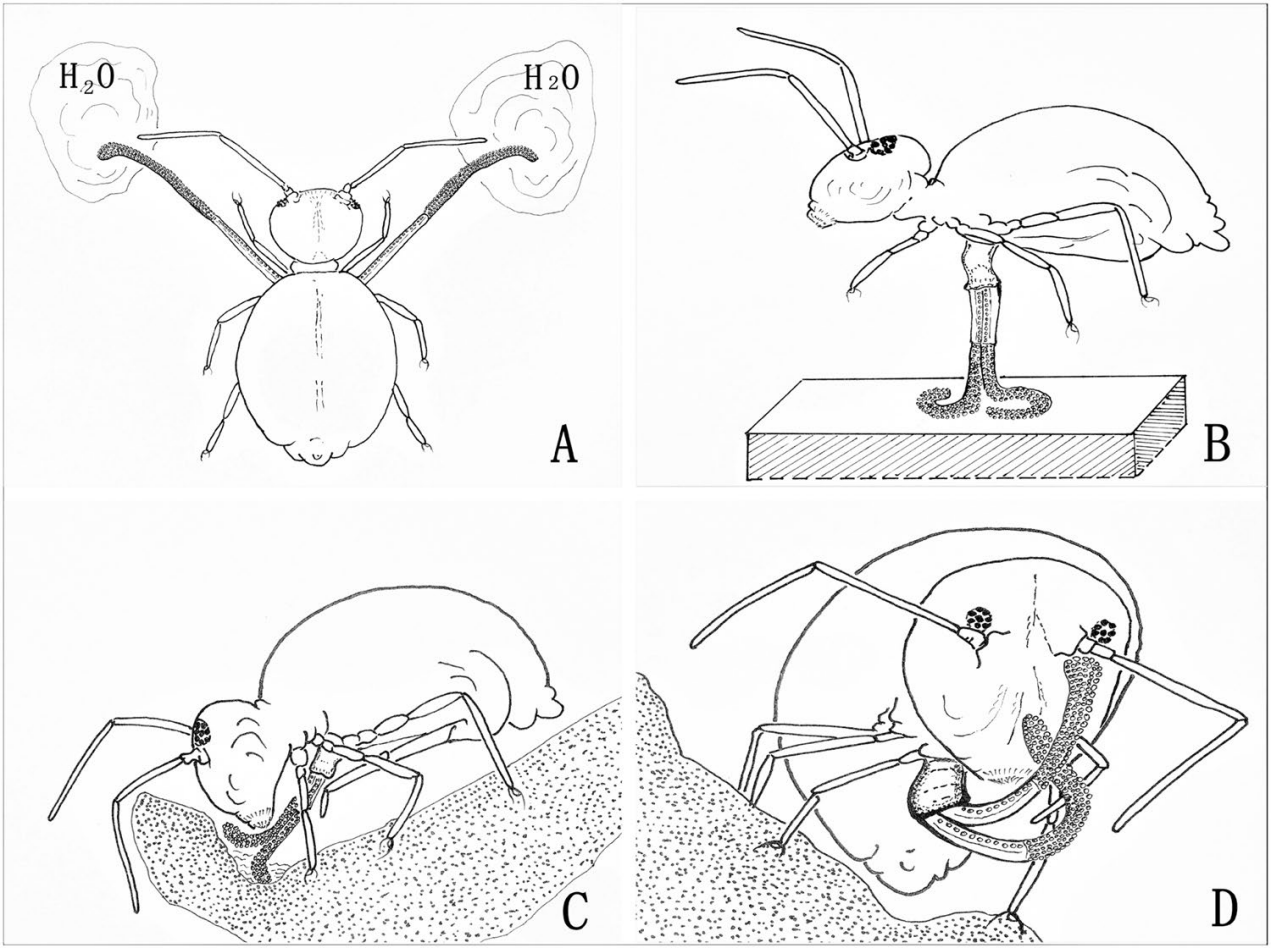
Extending motions of the eversible vesicles. (**A**) The eversible vesicles uptake water directly; (**B**) the springtail stands on the eversible vesicles in water; (**C**) the eversible vesicles help the mouthparts to uptake liquid food; (**D**) the eversible vesicles are cleaning the head. Scale bars = 0.1 mm.

Since the springtails inhabit the soil, it is necessary for them to frequently remove dusts or water droplets on its body and sense organs using the ventral tube. This behavior can be observed frequently under a macro lens. An important function of the eversible vesicles, when at rest, is to clean all part of the body surface. The most frequent cleaning part is the antennae (Fig. 4A,B). The eversible vesicles often extend one or two vesicles to clean their head, antennae, legs, tergum, and even anus (Figs 4A,B and 5D).

**Figure 6:**
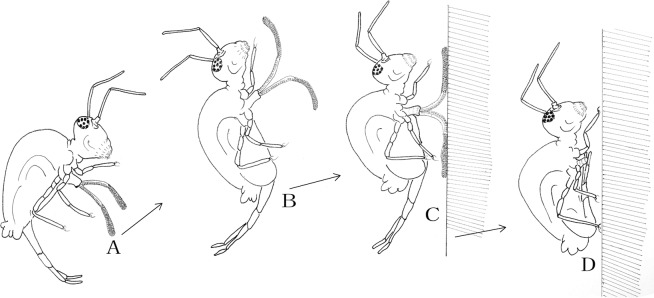
Jumping process of the springtail. (**A**) The eversible vesicles stretched out when the springtail started to jump; (**B**) the eversible vesicles fully extended; (**C**) the eversible vesicles adsorbed on a smooth surface when the springtail landed; (**D**) the eversible vesicles retracted into the body when the legs stood stable and the furca recovered under the abdomen.

The springtails are good at jumping. We used a hyaline plastic film at different heights (5, 10, 15 and 20 cm) to intercept the direction of their jumps. Each time it touched the plastic film, it would be preempted by the eversible vesicles and adhere to the smooth surface. When it started to take off, the eversible vesicles extended simultaneously. They stretched out the eversible vesicles at jumping, adsorbed on the substrate with the fully-extended reversible vesicles at landing, and retracted the eversible vesicles into the ventral tube immediately as soon as the legs stood stable (Fig. 6). The tiny papillae assisted the springtails to stay on a smooth surface. Numerous springtails can stay for a long period upside down on a glass (Fig. 4D). As a result, the eversible vesicles can ensure the safety and stability of their bodies in a complex environment (Figs 5B and 6).

The results of behavior observations show that the interceptions are roughly the same at different heights. For the majority of jumps, the eversible vesicles touched the transparent film first, and then adsorbed on the smooth surface. When the six legs were stable, the eversible vesicles can retract into the ventral tube.

## Discussion

After dissecting more than 100 samples of *S. viridis*, a relatively complete and clear slide specimen of the eversible vesicles was obtained. The presence of tiny papillae, transverse duct and central lumen confirms that the eversible vesicles are important organs of various functions. Because of the existence of small papillae, the eversible vesicles can be used to uptake water and liquid directly^3,4,6,8–12^. They are also a cleaning organ to clean the antennae and body surface^24^. At the same time, they are also a balance and stable organ, which allows the body to adhere to a smooth surface and maintain stability^1,2,9^. Previous morphological studies were mostly concentrated on the basal portion of the ventral tube for preserved dead specimens, thus were difficult to find the structure of the eversible vesicles^4,6–8^. Understandably, the eversible vesicles must be soft, flexible, and easy to control to perform various functions. The eversible vesicles are hyaline owing to the lack of pigments. The eversible vesicles are retracted into the ventral tube and body cavity most of the time. That is why the eversible vesicles are rarely observed in the specimens that are traditionally preserved and are observed with ordinary methods.

The eversible vesicles are a special organ with multiple functions, but are rarely found in other animals. To adapt to terrestrial life, the eversible vesicles cannot be extended out of the body all the time to make various necessary life activities. Alternatively, they should retract into the body when not use. The existence of small papillae and ducts in the eversible vesicles may partly explain the multifunctional behaviors of those springtails^27^.

The springtails are cleanliness-loving organisms, and do their cleaning work tirelessly when they keep immovable, repeatedly cleaning their heads, antennae, legs, terga and anus with the eversible vesicles. To prevent dust and debris from being carried on the body, the eversible vesicles need to be cleaned through a series of filtering and cleaning procedures, including a pair of forelegs, mouthparts and ventral tube bristles. The part most frequently cleaned is the antennae. Based on our observations, the springtails usually clean up their antennae more than 80 times per hour, 2–5 s each time.

During our present investigations, we also observed the internal structure of the ventral tube of the families Isotomindae and Tomoceridae in Collembola (data not shown). We found that the Sminthuridae have the longest eversible vesicles in Collembola. Other families have very short eversible vesicles, and some even have the ventral tube much more retracted in form. This indicates that the form of eversible vesicles may play a role in the classification and phylogenetic analyses of Collembola. The investigations of the fine structure of the eversible vesicles may also help understand the habits of the springtails and improve the integrative pest management of the Collombola^23^.

Admittedly, we observed the fine structure of the eversible vesicles only through light microscopy. More subtle ultrastructure should be found through scanning and transmission electron microscopy. In addition, the behavior observations are also without limitations. Evidently, more intense studies are needed to elucidate the structure and functions of the eversible vesicles in springtails.

## Methods

### Biological materials

Live springtails of *S. viridis* were obtained from the leaves of alfalfa on the north campus of the Northwest A&F University (34°28′N, 108°07′E, elev. 550 m), Yangling, Shaanxi Province in early April 2013. The springtails were reared in glass tubes or plastic boxes with fresh alfalfa leaves.

### Dissection and preparation of slide specimens

Live adult springtails were anaesthetized with diethyl ether and normal saline, and 20 individuals were dissected under an Olympus SZ61TR stereo microscope (Olympus, Tokyo, Japan). To effectively dissect the springtails, we made dissecting needles and a pair of tiny iris scissors by ourselves, and observed the springtails under a Leica DM500 biological microscope achromatic microlens equipped with a cold light illuminator (Leica, Wetzlar, Germany). Slide preparations followed standard procedures with a neutral optical resin adhesive and glutaraldehyde fixative, and stained with acid fuchsin and methylene blue, respectively. Photographs were taken using a SLR camera with a Nikon D750 + Macro lens under the Olympus SZ61TR stereo microscope. All the diagrammatic drawings were made based on our observation and photographs. Most behavioral observations were made under a camera with macro lenses.

### Behavior observations of the ventral tube

The water uptaking behavior of *S. viridis* through the ventral tube was recorded using a SLR camera with a macro lens at 26–30 °C.

For each experiment, 50 live springtails were placed in an incubator with a layer of soil about 3 cm thick or a dark colored paper at the bottom. The water content in the soil was less than 20%. When the springtails began to move around in search for food and water within an hour, we slowly injected 1 mL pure water into the soil or the paper. In most cases, most springtails moved quickly to the locality of moist soil, extended out an eversible vesicle, stayed in the soil for 3–6 s, and then moved to another location to repeat the activity. Video and photos were recorded from a transparent plastic box (26 cm × 18 cm × 16 cm). The experiment was replicated five times.

The high-speed video camera failed many times because of the small size of the springtails, its rapid jump and its random direction. We used a colorless transparent film to intercept different heights. Plastic film intercepted 5, 10, 15 and 20 cm above the springtail, and disturbed the springtails to force them to jump.
